# Extraction of Microbial and Host DNA, RNA, and Proteins from Oak Bark Tissue

**DOI:** 10.3390/mps2010015

**Published:** 2019-02-05

**Authors:** Martin Broberg, James E. McDonald

**Affiliations:** 1 School of Natural Sciences, Bangor University, Bangor LL57 2UW, UK; martin.broberg@helsinki.se; 2 Department of Biosciences, Faculty of Natural and Environmental Sciences, University of Helsinki, Biocenter 2, Viikinkaari 5, 00790 Helsinki, Finland

**Keywords:** oak tissue, oak bark, DNA extraction, RNA extraction, protein extraction, DNA/RNA sequencing, shotgun proteomics

## Abstract

The application of high-throughput nucleic acid and protein sequencing technologies is transforming our understanding of plant microbiomes and their interactions with their hosts in health and disease. However, progress in studying host-microbiome interactions in above-ground compartments of the tree (the phyllosphere) has been hampered due to high concentrations of phenolic compounds, lignin, and other compounds in tree bark that severely limit the success of DNA, RNA, and protein extraction. Here we present modified sample-preparation and kit-based protocols for the extraction of host and microbiome DNA and RNA from oak (*Quercus robus* and *Quercus petraea*) bark tissue for subsequent high-throughput sequencing. In addition, reducing the quantity of bark tissue used for an established protein extraction protocol yielded high quality protein for parallel analysis of the oak-microbiota metaproteome. These procedures demonstrate the successful extraction of nucleic acids and proteins from oak tissue using as little as 50 mg of sample input, producing sufficient quantities for nucleic acid sequencing and protein mass spectrometry of tree stem tissues and their associated microbiota.

## 1. Introduction

Advances in nucleic acid sequencing and proteomics technologies have revolutionized our ability to study the biology of plant hosts and their associated microbiota [1,2,3]. While the below-ground aspects of plant-microbiota interactions have arguably received greater attention, the importance of understanding the functions of hosts and their microbiota in above-ground components (phyllosphere) is becoming increasingly clear, particularly with respect to plant health and disease [2,3]. However, extraction of high-quality DNA, RNA, and protein from tree bark that comprises low biomass and numerous inhibitory phenolic and acidic compounds is a significant challenge and a major barrier to understanding host-microbiota interactions in the phyllosphere. Inhibitory compounds in oak bark are notoriously problematic to remove using proprietary nucleic acid extraction kits and procedures and are usually retained in the end-product eluate, resulting in nucleic acid degradation and inhibition of downstream analysis such as sequencing [4,5]. Here, we describe validated protocols for the extraction of DNA, RNA, and proteins from the oak caulosphere (stem tissue) for use in parallel host-microbiota analysis of Acute Oak Decline (AOD), a major threat to native British oak (*Quercus robur* and *Quercus petraea*) [6,7]. These procedures demonstrated the successful extraction of nucleic acids and proteins from oak tissue using as little as 50 mg of sample input, producing sufficient quantities for nucleic acid sequencing and protein mass spectrometry, and it is hoped they will be of value for scientists in the field. The procedures described here are cost effective, relatively uncomplicated, and avoid the use of hazardous chemicals like phenol and chloroform. These protocols were used to extract proteins and nucleic acids for analysis of the diversity and function of the oak microbiome in our previous studies of Acute Oak Decline, a complex Decline disease affecting native oak in the United Kingdom [6,7]. The advantages of the optimized methods described here relate to high extraction efficiency of high-quality DNA, RNA, and proteins from relatively low quantities of oak bark tissue (50 mg), a material that is difficult to work with. This was achieved through dilution of the bark tissue in larger volumes of buffer during the critical tissue maceration stage. This approach ultimately reduced the presence of inhibitors and enabled the generation of high yields of good quality nucleic acid and proteins for use in oak microbiome studies that have been a major barrier to understanding the composition and function of tree microbiomes. The DNA extraction focuses primarily on microbial (bacteria, archaea, viruses, and eukaryote) DNA, as this was of interest in our study of the pathogens involved with Acute Oak Decline. The mRNA is purified in this protocol in order to study gene expression profiles, and the procedures are simple and avoid hazardous chemicals such as phenol. In general, the techniques are designed to allow for the multi-omic analysis of trees and their associated microbiota, making them effective for taking forward the expanding use of multi-omics and host-microbiota interactions in tree disease research. 

## 2. Experimental Design

### Materials

- DNeasy Plant Mini kit (Qiagen, Venlo, Netherlands)
- Qubit dsDNA HS assay kit (Thermo Fisher, Waltham, MA, US)
- NEBnext microbiome DNA enrichment kit (New England Biolabs, Ipswich, MA, US)
- Genomic DNA Clean and Concentrator kit (Zymo Research, Irvine, CA, US)
- RNA extraction buffer (4 M guanidine thiocyanate, 0.2 M sodium acetate pH 5.0, 25 mM EDTA, 2.5% (*w*/*v*) polyvinylpyrrolidone, and 1% (*v*/*v*) β-mercaptoethanol)
- 20% sodium lauroyl sarcosinate
- RNeasy plant mini kit (Qiagen, Venlo, Netherlands)
- DNease I (Qiagen)
- RNeasy MinElute Cleanup kit (Qiagen)
- Ribo-Zero ribosomal RNA (rRNA) Removal kits for plant seed/root and for bacteria (Illumina, San Diego, CA, US)
- Qubit RNA HS assay kit (Thermo Fisher)
- Protein solubilization buffer (50 mM Tris-HCl, 25 mM EDTA, 500 mM thiourea, 0.5% DTT)
- Ice cold 20% trichloric acid in acetone with 0.5% DTT
- Ice cold acetone
- 3% SDS solution

## 3. Procedure

DNA extraction (run time: approximately 3 h; per sample cost: approximately £50, more than one extraction was required for some samples)

1. Use approximately 50 mg of oak tissue homogenized with a mortar and pestle under freezing conditions in liquid nitrogen. Transfer homogenized tissue into DNA extraction kit tube.
2. Use the DNeasy Plant Mini kit (Qiagen) according to the manufacturer’s instructions.
3. In order to enrich microbiome DNA, deplete the host DNA from the sample using the NEBnext microbiome DNA enrichment kit (New England Biolabs) according to the manufacturer’s instructions.
4. Purify the DNA and concentrate using the Genomic DNA Clean and Concentrator kit (Zymo Research) according to the manufacturer’s instructions.
5. Store at −20 °C.

RNA extraction (run time: approximately 4 h; per sample cost: approximately £185, based on a single RNeasy plant mini kit (Qiagen), RNeasy MinElute Cleanup kit (Qiagen), and Ribo-Zero ribosomal RNA (rRNA) Removal kit reaction (however, multiple columns were required for some samples)).

1. Bark samples collected for transcriptome analysis were immediately frozen in liquid nitrogen for on-site preservation of RNA. For RNA extraction, use approximately 50 mg of oak tissue homogenized with a mortar and pestle under freezing conditions in liquid nitrogen. Briefly, 5 mL of extraction buffer (4 M guanidine thiocyanate, 0.2 M sodium acetate pH 5.0, 25 mM EDTA, 2.5% (*w*/*v*) polyvinylpyrrolidone, and 1% (*v*/*v*) β-mercaptoethanol) is added to oak tissue kept frozen in a sterilized mortar using liquid nitrogen. The volume of extraction buffer used at this step was increased from the original protocol, as lower volumes of 1−2 mL were not sufficient to produce a significant quantity of RNA.
2. The frozen tissue in extraction buffer is further ground until thawed.
3. Add an additional 2.5 mL of extraction buffer and 500 μL of 20% sodium lauroyl sarcosinate mixed into the sample. Again, the volume added at this step has been increased from 60 to 500 μL.
4. Shake the sample mixture at room temperature for 15 min at 200 rpm on a shaking platform and further process using the RNeasy Plant Mini kit (Qiagen). The incubation time has been increased while the shaking intensity has been decreased in comparison to the original protocol.
5. After centrifugation in the QIAShredder column, mix 350 µL of the supernatant with 0.9 volumes of ethanol instead of 0.5 according to the kit protocol, and subsequently follow the manufacturer’s protocol by centrifuging in the RNeasy Mini column.
6. The manufacturer’s instructions for the RNeasy Plant Mini kit are followed from this stage onwards.
7. Treat the extracted RNA with DNase I (Qiagen) following the RNeasy Plant Mini kit (Qiagen) instructions for DNase treatment.
8. The RNA is purified using the RNeasy MinElute Cleanup kit (Qiagen) following the manufacturer’s instructions.
9. Deplete rRNA from RNA extracts using a 1:1 combination of the Ribo-Zero rRNA Removal kits for plant seed/root and for bacteria (Illumina) according to the manufacturer’s instructions.
10. Purify the rRNA depleted samples again using the RNeasy MinElute Cleanup kit (Qiagen) and store at −80 °C.

Protein extraction (run time: approximately 4 h, with one overnight incubation; per sample cost: approximately £15).

1. Use approximately 50 mg of oak tissue homogenized with mortar and pestle under freezing conditions in liquid nitrogen. A lower amount of bark tissue is used than in the original protocol (50 mg instead of 300 mg lyophilized powdered bark tissue) due to high levels of proteases and phenolic compounds.
2. Add 2 mL solubilization buffer (50 mM Tris-HCl, 25 mM EDTA, 500 mM thiourea, 0.5% DTT) and grind in a mortar and pestle at room temperature.
3. Shake the mixture on a platform shaker (150 rpm) for 1 h at ambient temperature.
4. Centrifuge samples at 20,000 g for 20 min and extract and store the supernatant at 4 °C. Repeat the procedure using the remaining pellet.
5. Extract the supernatant again and pool with the previous supernatant.
6. Add ice cold 20% trichloric acid in acetone with 0.5% DTT in a 1:1 ratio to the supernatant pool and precipitate at −20 °C overnight.
7. After precipitation, centrifuge the mixture at 20,000− *g* for 60 min and wash with ice cold acetone (centrifuged at 20,000− *g* for 30 min).
8. Air-dry the pellet, re-suspend in 3% SDS solution and store at −80 °C.

## 4. Expected Results

**Figure 1:**
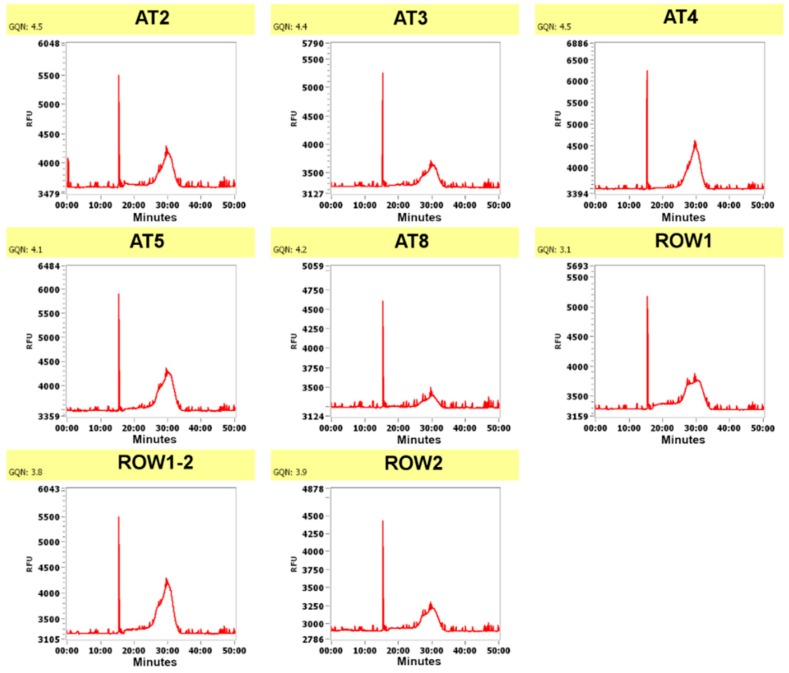
DNA fragment analyzer results for the samples using the PROSize v. 2.0 software. RFU signifies Relative Fluorescence Units (indicating the amount of DNA at a certain size/time), and the X-axis signifies time in minutes as the DNA fragments are separated during capillary electrophoresis.

The methods described here were used for our previous DNA/RNA sequencing and shotgun proteomic analysis of microbiota associated with healthy oak trees and oak affected by Acute Oak Decline [5,6]. The quantities of extracted nucleic acid or protein were determined using the corresponding Qubit HS assay kit (Table 1). DNA was extracted using the Qiagen DNeasy Plant mini extraction kit, and where host-DNA depletion was required for microbiome analysis, the New England Biolabs NEBNext microbiome enrichment kit was applied. Fragment analyzer analysis of all DNA samples revealed DNA of sufficient quality for sequencing using five bark samples from symptomatic trees (AT5, AT8, ROW1, ROW1-2, and ROW2) and three bark tissue samples from non-symptomatic trees (AT2, AT3, and AT4) (Figure 1, Table 2). 

**Figure 2:**
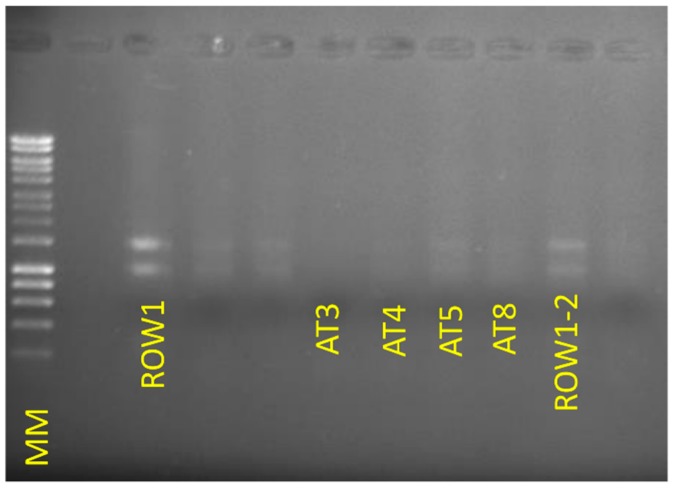
Agarose gel electrophoresis of RNA extractions from all samples in this study except for sample AT2. The molecular marker (MM) used was GeneRuler 1kb plus (ThermoFisher). For each sample, 6 µL of extracted RNA was loaded into each well. One sample, AT2, was not included in this electrophoresis gel.

**Figure 3:**
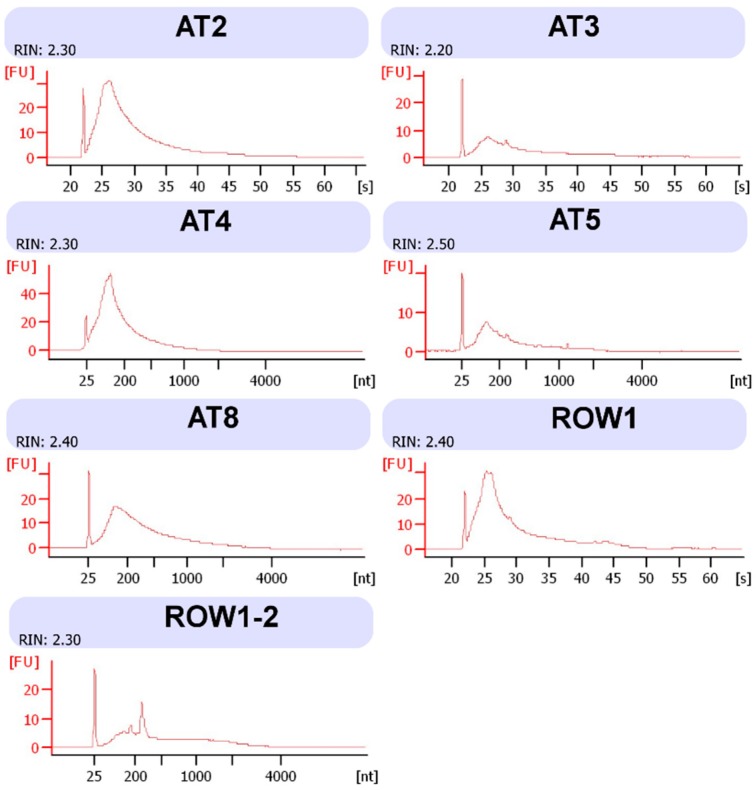
Bioanalyzer Eukaryote total RNA Pico graphs of all RNA extractions in this study. FU indicates Fluorescence Units, [s] indicates seconds and [nt] equals nucleotide size, which is interchangeable to seconds as larger nt fragments take longer to go through the gel.

For RNA extraction, we modified a protocol presented by Kalinowska et al. (2012) where a pre-extraction kit process is performed to remove inhibiting compounds, mixing bark tissue with a buffer containing polyvinylpyrrolidone, β-mercaptoethanol, and EDTA under freezing conditions [5]. The main differences in our protocol from the method in the key supporting paper concern a greater volume of buffer used and longer incubation steps with a higher shaking speed. Subsequently, the RNeasy Plant Mini kit (Qiagen) was used, and here our protocol contains some differences in the initial homogenization of oak tissue with the Qiagen Shredder column and mixing in ethanol. Furthermore, as we worked with sometimes macerated oak bark, we warn users to look out for this, as macerated dark brown oak bark severely reduces RNA yields in particular. However, the resulting yields of RNA were sufficient to allow for rRNA depletion and subsequent library preparation using the strand-specific ScriptSeq kit (Illumina) (Figure 2 and Figure 3). The protein extraction method is based on the protocol by Pragter et al. (2014), which utilizes an initial buffer of Tris, thiourea, and EDTA to solubilize the samples [8]. 

The resulting nucleic acid eluates (DNA and RNA) were successfully sequenced on the Illumina HiSeq 2500 platform, producing tens of millions of high-quality reads per sample (Table 2). The protein samples were successfully analyzed using the Tandem Mass Tag protocol on an Orbitrap Fusion Tribrid spectrophotometer. The only moment when it is imperative to work in a fume hood is during the use of β-mercaptoethanol, at the beginning stage of RNA extraction. The limitations of all extractions are the phenolic compounds of oak bark that may severely disrupt the procedures. Limitations may also include the amount of Qiagen tubes required for RNA extraction, as the volume of extraction buffer is quite large to sufficiently dilute the inhibiting compounds, resulting in the requirement for multiple (up to 10) spin columns for a single sample which are later pooled and concentrated. The Qiagen kit tubes may be re-used once for the same sample to reduce costs. Furthermore, adaptations of the protocols may be made in the quality of bark tissue, as macerated and degraded tissue is harder to work with, particularly when it comes to RNA. However, limitations would depend on the nature of the experiment. The protocol may be adjusted to handle a lower RNA extraction buffer volume by increasing the percentage of polyvinylpyrrolidone to soak up some phenols [9,10]. The protein extraction may be adjusted to target part of the proteome which is easily soluble in water, or insoluble membrane-associated proteins, depending on the alcohol ratios and centrifugation used. The New England Biolabs Next Microbiome DNA Enrichment Kit for host DNA depletion may be avoided if there is a desire to acquire host DNA as well. Thus, the protocols present some flexibility as to whether host or microbial DNA is the focus of downstream analysis. After sequencing and annotation of protein/nucleic acid read assembles, approximately 67−95% of associated sequence data in the metagenomes from symptomatic samples was of microbial origin (0.6−6% in non-symptomatic samples), 11−21% of sequence reads in the metatranscriptome from symptomatic samples was microbial (1−3% in non-symptomatic), and approximately 10% of data in the metaproteome from all samples was microbial [6].

## Figures and Tables

**Table 1:** Amounts of DNA/RNA/proteins after initial extraction.

**Sample Name**	**Total Amount (ng) DNA after Three Rounds of Extraction (Qubit dsDNA HS Assay)**
AT2	355
AT3	381
AT4	277
AT5-2	416
AT8	448
ROW1	293
ROW1-2	1334
ROW2	507
**Sample Name**	**Total Amount (ng) RNA after Two Rounds of Extraction (Qubit RNA HS Assay)**
AT2	2236
AT3	1725
AT4	1786
AT5-2	150
AT8	1551
ROW1	588
ROW1-2	5687
ROW2	Not successful
**Sample Name**	**Total Amount (ng) Protein (Qubit Protein Assay Kit)**
AT2	194
AT3	184
AT4	167
AT5-2	284
AT8	187
ROW1	212
ROW1-2	814
ROW2	248

**Table 2:** Sequencing results for all samples in amount of base pairs (bp).

Sample	DNA Sequencing bp	RNA Sequencing bp
AT2	6.60 × 10^9^	8.94 × 10^9^
AT3	5.57 × 10^9^	6.07 × 10^9^
AT4	7.04 × 10^9^	8.78 × 10^9^
AT5	6.66 × 10^9^	5.60 × 10^9^
AT8	6.98 × 10^9^	7.52 × 10^9^
ROW1	6.66 × 10^9^	8.97 × 10^9^
ROW1-2	6.42 × 10^9^	8.43 × 10^9^
ROW2	6.11 × 10^9^	Did not produce any
